# Supplementary material for: Patterns of Intron Gain and Loss in Fungi
Source: PLoS Biol. 2004 Nov 30;2(12):e422. doi: 10.1371/journal.pbio.0020422 (PMC532390; doi:10.1371/journal.pbio.0020422)
Supplement: Table S1 — Also available at http://genes.mit.edu/NielsenEtAl/. (4.3 MB ZIP). [file pbio.0020422.st001.zip › NielsenEtAl/html/741.html]

AN6171.1.NCU01008.1.MG04201.1.FG10219.1


```
 CLUSTAL W (1.82) Multiple Sequence Alignments - Introns Inserted


Sequence 1: NCU01008.1	183 aa
Sequence 2: FG10219.1	183 aa
Sequence 3: MG04201.1	183 aa
Sequence 4: AN6171.1	188 aa
Alignment Length: 188 aa
Number Identitical Residues: 97 aa
Alignment Score (without introns) 4792


MG04201.1 	MVRKLKHHEQK2LLKKVDFVTYKGDNDHRAAAVCR~RYMIQKPSDY~HTYNQICG~SLRK
NCU01008.1	MVRKLKHAEQK~LLRKTDFINYKSDNKHRDHDVAR2RYMIQKPEDY0HKYNRMCG0SLRQ
FG10219.1 	MVRKLKHHESK~LLRKTDFITYKGDNGHRDKAVMR~RYMIQKPEDY~HKYNRLCG0SLRQ
AN6171.1  	MVRKLKHHEQK2LLRKVNLTTYKSDHAHRESQIIQ~RYYLQNPVDY~KKYSAIVG~SLRQ
          	******* *.* **:*.:: .**.*: **   : : ** :*:* ** :.*. : * ***:

MG04201.1 	LAHRLSLLPPDSE-VRRKHEALLLEKLYDVGVLS~TQSK----LSAVEHGATVSAFARRR
NCU01008.1	LAHRLSLLPPDNE-VRRKHEELLLDKLYDMGILS0SKSK----LSQVEHQVTVSAFARRR
FG10219.1 	LAHRLSLLPPENA-TRRKHEELLLNKLYDMGVLS~SSSK----LSAVENSVTVSAFARRR
AN6171.1  	LAHKLSALDPDSDPIRQKTESEVLDKLWRMGILK~QSREQGAGLSRIEREVTVSAFCRRR
          	***:** * *:. . *:* *  :*:**: :*:*.  . :..:.** :*. .*****.***

MG04201.1 	LPVVMTRLHMAENIST~ATKLIEQGHVRVGVDVVTDPAMLITRSMEDFVTWAVGSKIKKN
NCU01008.1	LPVVMTRLRMAETVQA~ATKMIEQGHVRVGVEEVRDPAFLVTRSMEDFVTWSVGSKIKQN
FG10219.1 	LPVLMTRLRMAETVQA~ATKMIEQGHVRVGTETVTDPAYLVTRGMEDFVTWTVGSKIKRN
AN6171.1  	LAVVMARSGMVENIKT0AVTFIEQGHVRVGSEVVTDPAYLVTRNMEDFVTWVDSSKIKRN
          	*.*:*:*  *.*.:.: *..:********* : * *** *:**.*******  .****:*

MG04201.1 	IMKYKDKLDDFELL
NCU01008.1	IMKYRDKLDDFELL
FG10219.1 	IMKYRDQLDDFELL
AN6171.1  	IMRYRENLDDFDLM
          	**:*:::****:*:
```
